# Computational spirits: a neuroscientific account of psychedelic entity encounters

**DOI:** 10.1093/nc/niaf069

**Published:** 2026-06-05

**Authors:** Jonas Mago, George Deane, Lars Sandved-Smith, Adam Safron, Christopher Timmermann, Maxwell Ramstead, David Dupuis, Robin Carhart-Harris, Kyle T Greenway, Michael Lifshitz

**Affiliations:** Integrated Program in Neuroscience, McGill University, Room 302, Irving Ludmer Building 1033 Pine Ave. W. Montreal, Quebec H3A 1A1, Canada; Division of Social and Transcultural Psychiatry, Department of Psychiatry, McGill University, 1033 Pine Ave Montreal, Quebec, Canada; Lady Davis Institute for Medical Research, Montreal Jewish General Hospital, 4333 Chem. de la Côte-Sainte-Catherine, Montréal, QC H3T 1E4, Canada; Department of Philosophy, University of Montreal, Pavillon 2910, boul. Édouard-Montpetit Montréal QC H3C 3J7; Monash Centre for Consciousness and Contemplative Studies, Monash University, 29 Ancora Imparo Way, Clayton, VIC, 3800, Australia; Allen Discovery Center, Tufts University, 200 Boston Avenue Medford, MA 02155, USA; Institute for Advanced Consciousness Studies, 2811 Wilshire Blvd # 510, Santa Monica, CA 90403, United States; SapiensAI; DMT Research Group, Centre for Psychedelic Research, Division of Psychiatry, Department of Brain Sciences, Imperial College London, South Kensington Campus, London SW7 2AZ, UK; Division of Social and Transcultural Psychiatry, Department of Psychiatry, McGill University, 1033 Pine Ave Montreal, Quebec, Canada; Queen Square Institute of Neurology, University College London, Gower Street, London, WC1E 6BT, UK; Noumenal Labs, P.O. Box 670403, Dallas, TX 75367, USA; Institut de recherche interdisciplinaire sur les enjeux sociaux (EHESS, Paris) / INSERM, Bâtiment recherche Sud, 5 Cr des Humanités, 93300 Aubervilliers, France; Centre for Psychedelic Research, Division of Psychiatry, Department of Brain Sciences, Imperial College London, South Kensington Campus, London SW7 2AZ, UK; University of California San Francisco, 1001 Potrero Ave, San Francisco, CA 94110, United States; Division of Social and Transcultural Psychiatry, Department of Psychiatry, McGill University, 1033 Pine Ave Montreal, Quebec, Canada; Lady Davis Institute for Medical Research, Montreal Jewish General Hospital, 4333 Chem. de la Côte-Sainte-Catherine, Montréal, QC H3T 1E4, Canada; Division of Social and Transcultural Psychiatry, Department of Psychiatry, McGill University, 1033 Pine Ave Montreal, Quebec, Canada; Lady Davis Institute for Medical Research, Montreal Jewish General Hospital, 4333 Chem. de la Côte-Sainte-Catherine, Montréal, QC H3T 1E4, Canada

**Keywords:** active inference, DMT, psychedelics, computational modeling, phenomenology

## Abstract

Under the influence of psychedelics, people often report encountering “entities” who seem to have their own autonomous agency. Depending on the cultural milieu, these entities are reported to take a variety of forms, including spirits, elves, ancestors, or fragments of the self. Encounters with such beings hold a central place in many traditions of psychedelic practice around the world. And yet, mechanistic accounts of these experiences are scarce in the neuroscientific literature. Here, we propose a neurocomputational model to account for experiences of entities, focusing primarily on those occasioned by the serotonergic psychedelic N,N-dimethyltryptamine. Our model builds on earlier theoretical accounts, including the entropic brain model of psychedelics, computational accounts of the felt presence of other minds, and theories of self-other discrimination based on sensory attenuation. We synthesize and expand on these perspectives through an overarching physics-based approach to cognition and brain function—the active inference framework. We propose that the general effects of psychedelics on large-scale neural dynamics may shape the way the brain comes to infer and interpret agentic presences. In particular, the reduction in the predictability of sensory perceptions during the psychedelic state may incline the brain to interpret perceptions, both internal and external, as resulting from non-self-agentic sources. In specifying the neurocomputational mechanisms, our model aims to explain how the brain supports entity encounters while also accounting for the diversity (and similarity) of these experiences across cultural contexts.

## Introduction

Psychedelics are known to profoundly reconfigure the sense of self and self-other boundaries. For example, experiences of "ego-dissolution" have been studied extensively in the scientific literature on psychedelic substances. However, rather than the dissolution of the sense of self, another set of psychedelic experiences involves encountering novel presences, or entities, who are experienced as having their own autonomous agency. Such entity experiences can be occasioned by a range of compounds, including classic serotonergic psychedelics, such as psilocybin, lysergic acid diethylamide (LSD), mescaline, and N,N-dimethyltryptamine (DMT), as well as non-classic psychedelics including ketamine, salvia, ibogaine, and Amantia muscaria (Meyer 1994; Luke 2011; Davis et al. 2020; Luke et al. 2021; Lutkajtis 2021; Michael et al. 2021; Lawrence et al. 2022; Nayak and Griffiths 2022). This paper proposes a neurocomputational model to capture some of the key phenomenological features of entity encounters shared across psychedelics.

We take active inference as our guiding theoretical framework. Active inference is a physics based approach to modeling living systems. It describes how agents develop internal models of themselves and the world and update those internal models by performing actions and receiving sensory feedback from their environment (Friston 2010, 2012, 2019). Previous research has used active inference and related approaches (i.e. the free-energy principle) to model many of the phenomenological effects of psychedelics, including the experience of ego-dissolution (Carhart-Harris and Friston 2019; Deane 2020; Deane et al. 2020). Here, we extend these models to explain how alterations in the brain mechanisms underwriting the sense of self can lead to psychedelic experiences involving encounters with entities. As a neuroscientific framework, our model does not take a stance as to the ontological nature of entities, but rather aims to account for the subjective experience of encountering them.

## An introduction to psychedelic entities: from neurobiology to cultural phenomenology

Entity encounters can range from a vague feeling of presence to a belief-shaking meeting with a spirit, god, ancestor, or inner child. The phenomenology of these experiences differs along various dimensions, including the degree to which the entity is personified, how much it is perceived in different sense modalities, its affective valence, and the feeling of its intention towards the perceiver. Such phenomenological diversity is reflected in the wide variety of names attributed to entities. The literature is replete with accounts of spirits, guides, gods, aliens, elves, angels, faeries, demons, gnomes, dwarves, and ancestors (Winkelman 2018; Griffiths et al. 2019; Davis et al. 2020). The entities encountered can also consist of aspects of the self, including those that have been neglected or forgotten (Watts et al. 2022).

Among indigenous groups, anthropologists have repeatedly observed the use of psychedelics to connect with supernatural entities (Harner 1973; Furst 1976; Dobkin de Rios 1984). For example, in the Western Amazon, ayahuasca (a traditional DMT-containing brew) is offered during shamanic initiations or rites of passage to help participants encounter supernatural beings such as nature spirits, ancestors, and mythological figures (Harner 1972; Reichel-Dolmatoff 1972; Kensinger and Harner 1973; Descola 1996; Shanon 2002; Déléage 2009). The quest for encounters with supernatural entities (such as the “spirit of ayahuasca”) is also one of the main motives that leads tourists from the Global North to travel to the Amazon to participate in shamanic-inspired rituals. Such "shamanic tourism" has increased significantly in the Peruvian Amazon over the past few decades (Winkelman 2005; Dupuis 2022a).

While entity encounters are central to many indigenous and syncretic forms of psychedelic practice, they are also common in contemporary recreational and even medical contexts (Richards 2015; Griffiths et al. 2019). Similar experiences also hold an important place in spiritual practices that do not employ psychedelic substances, such as traditions of active imagination, mediumship, prayer, and meditation (Luhrmann et al. 2023; Powers et al. 2017; Seligman and Kirmayer 2008). In these practices, people deliberately train their attention to cultivate sensory and imaginative relationships with invisible others such as gods and spirits. Research on evangelical Christian prayer, for example, shows that people can train themselves to audibly hear the voice of God (Luhrmann 2012; Luhrmann and Morgain 2012; Luhrmann et al. 2013; Luhrmann et al. 2021).

Experiences of non-self-presences are, of course, common among patients with a variety of clinical conditions including forms of dissociative identity disorder, psychosis, Charles Bonnet Syndrome, and dementia (Merckelbach et al. 2002; Grover et al. 2014; Powers et al. 2018; Alderson-Day, Moseley, et al. 2021; Alderson-Day, Woods, et al. 2021b; Blom 2021; Le Blanc 2022). In contrast to the deliberate cultivation of entity experiences through spiritual or psychedelic practice, clinical experiences of non-self-entities typically occur involuntarily and tend to cause distress (Johns et al. 2014; Luhrmann et al. 2019).

Across all of these cases, the specific form and content of the sensed presences will vary substantially according to the individual personality, beliefs, and history of the experiencer, as well as the broader sociocultural contexts in which they are embedded (Seligman and Kirmayer 2008; Luhrmann et al. 2015a, 2015b, 2021; Lifshitz et al. 2018, 2019; Dupuis 2022b). Comparative studies of the uses of psychedelics show that, even if some features of the experiences are similar across contexts—such as geometric patterns reflecting structural features of the occipital system (Cowan 2013) or social figures reflecting common human concerns), most features vary extensively depending on the cultural milieu (e.g. feeling-tone, meaning, or form of the visions). Many ethnographers have observed homogeneity in the content of the psychedelic experience within the same culture, which has led them to defend a culturalist approach (Mooney 1896; Wallace 1959; Lévi-Strauss 1970; Dobkin de Rios 1972; Reichel-Dolmatoff 1972; Brown 1978; Langdon 1979). For instance, terms such as “culturally influenced visions” (Langdon 1979) or “stereotypic visions” (Dobkin de Rios 1974) have been used to emphasize the role of cultural variables in the psychedelic experience. Some have argued that the mind-altering properties of psychedelic substances make them ideal for building and maintaining social affiliation and cohesion. As they are deeply influenced by cultural background and social interactions, psychedelic experiences can serve as powerful vectors of belief transmission and consolidation of social groups (Pace and Devenot 2021; Dupuis and Veissière 2022). This aligns with clinical case studies showing that recent media exposure can influence, or “imprint,” the content of psychedelic imagery (Garel et al. 2023). Similarly, participants in psychedelic rituals often encounter the kinds of entities that conform to local cultural invitations (Dupuis 2021; Nayak and Griffiths 2022). Nonetheless, many psychedelic users report being surprised when encountering entities for the first time, especially in contexts that lack a strong cultural frame for such experiences (Timmermann et al. 2021).

Despite the diverse nomenclatures and phenomenologies associated with entity encounters, there also seems to be some consistency in the features of these experiences across the different contexts in which they occur (Winkelman 2018; Griffiths et al. 2019). In an online survey of mostly white, male, North Americans who had experienced an encounter with God or a higher power, Griffiths et al. (2019) asked participants about their “most memorable God encounter experience”. They collected thousands of reports, including psychedelic as well as non-drug experiences. The results suggest that people commonly experience the encountered beings as conscious and intelligent and as existing in some real but different dimension of reality. Many who experienced such encounters reported that they were among life’s most meaningful experiences, regardless of whether they were experienced in a religious or secular context.

While entity encounters can be induced by various psychedelic substances (Beach 1996; Shanon 2002; Nayak and Griffiths 2022), they are particularly common under the influence of DMT (Strassman 2000; Luke et al. 2021). DMT is a naturally occurring substance in plants, animals, and the human brain. In the mid-1990s, Richard Strassman conducted the first laboratory study of DMT (Strassman 1995, 2000). He found that, among 60 participants receiving a total of over 400 doses of DMT, half of the participants administered the high doses (21–28 mg/kg, I.V.) reported encountering entities. More recently, a study interviewing DMT users after their recreational dosing sessions showed that 34 out of the 36 DMT experiences involved an encounter with some form of sentient entity who was experienced as “beyond the self” (Michael et al. 2021, 2023). The study furthermore used inductive thematic analysis to reveal that DMT-elicited entities were often perceived as helpful (in 53% of the reports) or communicative (47%) and ranged in demeanor from inviting (56%) to fearsome (8%).

Large-scale online survey data further support the prevalence of entity encounters under DMT. A study of 3 778 DMT trip reports (mostly from males, with no report of ethnicity) posted to the Reddit community, r/DMT, found that 45% of these reports mentioned experiences of entities. These were referred to by a variety of names, including deities (17%), aliens (16%), creature-based entities like reptiles or insectoid beings (9%), mythological beings including “machine elves” (8%), and jesters (7%) (Lawrence et al. 2022). Another recent online survey gathered responses from 2 561 people (largely white males from the US) who reported entity experiences occasioned by DMT, and found that the most prominent emotions associated with these experiences were love, kindness, and joy, although 41% of respondents also reported experiencing fear. Many respondents reported receiving a message (69%) or a prediction about the future (19%) and more than half of those who identified as atheist before the experience no longer identified as atheist afterwards (Davis et al. 2020).

Even brief entity encounters can have lasting effects on metaphysical beliefs. One survey investigated changes in beliefs longitudinally following a ceremonial psychedelic session (Timmermann et al. 2021). This study recruited 866 participants (mostly white, US or UK-based, of balanced gender) who were planning to attend a psychedelic ceremony in the near future (using psilocybin, ayahuasca, DMT, San Pedro, or LSD). They were asked to describe their metaphysical beliefs one week before the ceremony, and then again four weeks and six months later. Results showed that a single psychedelic experience caused significant shifts in beliefs away from “physicalist” or “materialist” views toward more “panpsychist” or “fatalist” views. The shift toward panpsychism, i.e. the perspective that all things have a mind or mind-like quality, endured at 6 month follow-up. This shift occurred for more than half of the respondents who previously identified as agnostic on the matter. Along similar lines, another recent study surveyed participants who endorsed having a belief-changing psychedelic experience (*n* = 1606, including mostly white, US-based males) (Nayak and Griffiths 2022). This study found that following the experience, participants were more likely to attribute consciousness to a range of living and non-living entities, including non-human primates (63%–83%), quadrupeds (59%–79%), insects (33%–57%), fungi (21%–56%), plants (26%–61%), inanimate natural objects (8%–26%), and inanimate manmade objects (3%–15%). These findings show that psychedelic experiences can profoundly alter perception and metaphysical beliefs, including by fostering a tendency to attribute consciousness to various kinds of non-human beings. In this paper, we propose a computational model that may help to explain why psychedelics predispose people to infer the presence of agentic, sentient others in ambiguous cases such as these.

Symbolic and thematic motifs seem to recur in psychedelic experiences across cultures, such as the experience of meeting a benevolent guide. This has led some scholars and practitioners to consider these experiences through the lens of Jungian archetypes (Brouwer et al. 2024; McGovern et al. 2023; McGovern et al. 2025; Richards 1978, 2002). Jung’s understanding of archetypes shifted over the course of his career, but he tended to describe them as universal, primordial patterns within the human psyche that structure our experience and take shape imagistically, especially in dreams, myths, and liminal states of consciousness (Jung 1959; Jung and von Franz 1964). More recent accounts typically describe archetypes as latent conceptual forms, passed down through evolution, which manifest in specific forms depending on the particular life experience and cultural background of each individual (Kirmayer 1993; Hogenson 2019; Roesler 2019). This view is broadly aligned with earlier work in biogenetic structuralism, which proposed that such psychological structures are grounded in inherited neurocognitive templates (Laughlin and d'Aquili 1974).

Although the concept of the archetype remains controversial, most people would agree that many significant human experiences are universal, such as being born, being mothered, falling ill, or encountering a non-human animal. Moreover, many of the most salient of these universal experiences seem to be social in nature, perhaps because human beings, like other mammals, rely heavily on social attachments from an early age. It seems plausible that these common human encounters have shaped the inherited affordances of our nervous system and imagination, predisposing human brains to generate images that reflect these universal life experiences (Kirmayer 1993; Winkelman 2018). A few recent mechanistic accounts have attempted to explain how these inherited motivational (and often social) affordances interact with our brain’s capacity for mental imagery, memory, and cultural learning to manifest as individually-specific archetypal imagery (McGovern et al. 2023; Vaughn Becker and Neuberg 2019).

Entity encounters, with their often powerful emotional resonance and symbolic meaning, could be seen as the manifestation of such archetypal forms, brought to the surface through the altered states of consciousness induced by psychedelics (McGovern et al. 2025; Richards 2002). As Szára—one of the first researchers to study DMT—suggested, these forms may originate “right in the brain,” encoded in neuronal connectivity patterns laid down early in development and released into perception through the disinhibitory effects of DMT (Gallimore and Luke 2015). However, some anthropologists have criticized this return to the concept of archetypes (Barrett 2019), questioning whether the construct remains useful in light of a sophisticated body of research that has emerged since Jung’s time on cross-cultural concept formation (e.g. Sperber and Hirschfeld 2004; Barrett 2015; Veissière et al. 2020). This critique centers on whether a meaningful distinction can be drawn between archetypes and other cross-cultural concepts—such as *dog*, *sister*, or *hand—*which, like archetypes, derive from (nearly) universal human experiences and are likely to evoke imagery based on each individual’s life history (Barrett 2019). In any case, whether or not we want to call them archetypal, it seems clear that psychedelic entity encounters show both striking commonalities across contexts (potentially rooted in the evolved social affordances of our imagistic systems), as well as marked differences in their specific manifestations among particular individuals and cultural environments.

## Towards a neuro-computational account of psychedelic entity encounters

In this paper, we aim to develop a neurocomputational model of entity encounters to explain how the effects of psychedelic substances on brain function may propel these powerful experiences of agentic presence. Previously, Michael Winkelman (Winkelman 2017, 2018) proposed a neurophenomenological account of entity experiences founded in evolutionary psychology (Gardner 2011; Gardner 2000). According to this evolutionary approach, human cognition can be understood as an assembly of evolved modules, each specialized through natural selection to perform particular cognitive functions. While modularist approaches to the study of cognition have been criticized, particularly in light of findings demonstrating the complex distribution of cognitive processing throughout the brain (Woodward and Cowie 2004), recent theoretical work suggests that the apparent modular organization of brain function may in fact reflect a statistical property of factorization rather than genuine anatomical segregation, arising from the mean-field dynamics of hierarchically coupled inferential processes (Parr et al. 2020). We nevertheless take inspiration from Winkelman’s proposal that psychedelic states may accentuate an evolved bias of the human brain towards attributing agency when sensory input is ambiguous. Indeed, even in infancy, the human brain has special systems dedicated to perceiving faces and detecting biological motion, both of which reflect an inborn proclivity toward modeling other minds and discerning intentionality (Simion et al. 2011). Here, we wish to contribute to the work on entity experiences by proposing a model rooted in a computational approach towards cognition, specifically leveraging the active inference framework. While the modularist approach highlights the functional specialization of cognitive processes, active inference can be considered as an alternative and complementary framework that explains how integrated, hierarchical, and dynamically interacting processes continuously update to minimize prediction errors and thereby generate perceptions and actions. In this framework, the brain is understood as a hierarchically-nested inferential system: a multilevel architecture in which lower levels process immediate sensory data while higher levels generate increasingly abstract predictions—about bodies, minds, and social agents—that are continuously refined *via* feedback from prediction errors (Kiebel et al. 2008; Friston 2010; Bastos et al. 2012). A similar development building on the idea of an evolved modular agency detection bias is offered by Andersen et al. (2019), who frames agency detection within a predictive processing framework. He argues that when prior expectations are strong but sensory precision is low—as often occurs in psychedelic states—the brain is more likely to generate false positives, both in terms of whether an agent is perceived and in terms of the agent’s specific perceptual features. Embedded in the active inference framework, our model aims to account for several key phenomenological characteristics of entity encounters: namely, (i) the feeling of non-self-agentic presence, (ii) the personification of this presence, and (ii) the divergent characteristics of entity experiences across cultures, individuals, sets, and settings.

### DMT and relaxed beliefs under psychedelics (REBUS)

Many psychedelics, and even non-pharmacological practices, can facilitate entity encounters. Here we will focus on DMT because it seems to be one of the most reliable ways to have such an experience (Meyer 1994; Luke 2011; Davis et al. 2020; Michael et al. 2021; Lawrence et al. 2022). However, we believe that our model likely holds true for other psychedelics, and may also inform our understanding of non-pharmacological entity encounters.

DMT falls into the category of serotonergic psychedelics, sometimes referred to as “classic” psychedelics, along with other substances such as LSD and psilocybin. Serotonergic psychedelics act on a specific serotonin receptor subtype, the 5-HT2AR (Ra et al. 1992; Vollenweider et al. 1998; Timmermann et al. 2023). The 5-HT2ARs are expressed throughout the human brain, but they are most densely expressed in the primary visual cortex as well as in association networks related to high-level cognition, such as the default mode network (DMN) (Beliveau et al. 2017). These are therefore the areas that are impacted most by serotonergic psychedelics, including DMT, LSD, and psilocybin (Vollenweider et al. 1998; Vollenweider and Kometer 2010; Carhart-Harris et al. 2012, 2016; Timmermann et al. 2023; Siegel et al. 2024).

The DMN is an association network that is densely interconnected with much of the brain. Key nodes of the DMN include the medial posterior cortex (specifically the posterior cingulate cortex; PCC and parts of the precuneus), the medial prefrontal cortex, bilateral inferior parietal lobule, and the temporo-parietal junction (TPJ) (Hagmann et al. 2008). Some researchers propose that the DMN sits at the top of a hierarchical processing system, by which information from different brain regions and sensory modalities is integrated (Margulies et al. 2016; Friston 2018; Friston et al. 2018). The DMN generally plays an important role in high-level abstract processing and metacognition (Soto et al. 2018), and more specifically in mental time travel (Østby et al. 2012), moral decision-making (Kaplan et al. 2017), counterfactual thinking (Van Hoeck et al. 2013), self-consciousness (Fingelkurts et al. 2012), and social cognition (Yeshurun et al. 2021).

The dysregulation of activity in the DMN and other high-level association cortex during the psychedelic experience has inspired a predictive processing model referred to as “RElaxation of Beliefs Under pSychedelics” or “REBUS” (Carhart-Harris and Friston 2019). This model suggests that the increased entropy in global brain activity patterns (especially but not exclusively within the DMN and other high-level association cortex) parallels a relaxation (or decreased “precision-weighting”) of the brain’s predictive models of self and world. Dysregulating high-level neural activity that ordinarily encodes high-level cognitive models and has a constraining influence on global brain function, enables ordinarily constrained activity to operate relatively freer of such constraint— translating into a richer quality of conscious experience (Friston 2010; Carhart-Harris et al. 2014, 2016; Carhart-Harris 2018; Carhart-Harris and Friston 2019). Accordingly, the relaxing of predictive models encoded *via* the regularities and hierarchical organization of spontaneous brain activity (where predictive models or “priors” are named “beliefs”) under psychedelics may explain the increased richness (i.e. the diversity and vividness) of subjective experience (Hilgetag and Hütt 2014).

In active inference, beliefs are encoded as prior expectations over hidden causes of sensory input, organized hierarchically across neural systems. These beliefs function to constrain interpretation, reducing uncertainty and ensuring perceptual stability. Psychedelics are hypothesized to reduce the precision particularly of high-level priors, thereby increasing bottom-up influence and allowing otherwise suppressed or discounted sensory and affective data to emerge. This may result in an increased need to model surprising sensory content, which under certain conditions may be resolved by inferring the presence of autonomous agents.

Recent neuroimaging evidence supports the idea that DMT relaxes the influence of high-level beliefs/models in the brain. Timmermann et al. (2019) found that intravenous (IV) DMT increased spontaneous signal diversity (a measure formally related to entropy) of brain activity determined with EEG. In a more recent study from the same group, this time employing simultaneous EEG-fMRI, DMT-induced increases in signal diversity were found to correlate with disruptions of connectivity in high-level networks (including the DMN), as well as with ratings of “richness of experience”. Furthermore, DMT was shown to specifically reduce the integrity and segregation of these brain networks, while increasing hyperconnectivity between these systems and the rest of the brain (Timmermann et al. 2023). Similar findings of reduced functional network segregation, increased signal diversity, and hyperconnectivity of higher-order association networks have been reported with other serotonergic substances including psilocybin and LSD (Roseman et al. 2014; Carhart-Harris et al. 2016; Tagliazucchi et al. 2016; Girn et al. 2022; Mortaheb et al. 2024). These brain patterns have been related to some of the most striking changes in phenomenology induced by psychedelics, including experiences of self-transcendence, ego-dissolution, and oceanic boundlessness (Tagliazucchi et al. 2016; Mason et al. 2020; Siegel et al. 2024). However, it is important to note that in some studies, the observed hyperconnectivity induced by serotonergic psychedelics has been located in primary sensory areas rather than transmodal areas (Preller et al. 2018, 2020).

### 1.1. Active inference and the sense of self

We now introduce active inference as our theoretical and modeling framework, and revisit the experience of encountering entities from a computational perspective. Active inference is an increasingly popular computational approach to modeling physical phenomena, and is especially useful when modeling systems that make decisions under uncertainty—i.e*.* agents. The active inference toolkit enables us to write down tractable computational models of how agents update their internal models of the world and select relevant actions in situations of uncertainty. In this paper, we do not develop a formal mathematical implementation, but instead leverage the theoretical structure of active inference to conceptually model the processes involved in psychedelic entity encounters. From this perspective, agents are cast as needing to infer the state of the world, and needing to infer what to do next, on the basis of partial observations and in the face of volatility and noise. Living organisms continuously adapt to their environment by optimizing evidence for probabilistic models of the world. These “generative” models are structured hierarchically, from low-level sensory perceptions, such as the detection of object edges, to high-level abstract beliefs, such as inferences about the intentions of other agents (Friston 2010, 2019; Hohwy 2016; Friston 2012).

Within the active inference framework, the sense of self or “phenomenal selfhood” has been modeled using an “allostatic control model” (Deane 2020, 2022; Deane et al. 2020). In this view, the sense of being a self-arises when a system is equipped with the ability to make inferences about its own “endogenous” capacity to control sensory outcomes, contingent on its own actions. This is called an allostatic control model, where allostasis is understood as the *predictive regulation* of bodily states. Whereas in homeostasis, autonomic reflexes react to external changes in the environment to return the organism to homeostatic setpoints, allostasis involves maintaining the integrity of the organism by preemptively modifying internal states in anticipation of likely changes in the environment. According to this allostatic control view, the feeling of being a self-arises from the system *modeling itself* as the principal “endogenous cause” or “controller” of survival-relevant sensations (Frith 2000; Haggard and Tsakiris 2009; Hohwy and Michael 2017; Allen and Friston 2018).

The organism’s allostatic control model (i.e*.* self-model) is understood to be hierarchically structured. Minimal embodied aspects of selfhood are associated with attenuation processes in lower levels of the allostatic control model, closer to the sensory end of the hierarchy. For example, the basic feeling of being an agent emerges through attenuation of “reafferent” inputs, i.e*.* inputs generated by the organism's own movement (Jékely et al. 2021)*.* In simple organisms, this is accomplished *via* automatic sensory attenuation mechanisms—for example, movement will dampen incoming sensory inputs that are predicted as a consequence of that specific action. In more complex organisms, when the brain sends a motor command to the muscles, a copy of those signals is also sent to other brain regions. These reafferent signals, referred to as corollary discharge, prepare the sensory areas for impending movement, enabling them to distinguish sensory inputs caused by one's behavior from those caused by the external world (P. Corlett et al. 2019; Crapse and Sommer 2008). These basic allostatic control processes support the minimal, embodied feeling of being an agentic self.

Within this active inference account, such minimal, embodied models of selfhood/allostatic control become integrated over space and time into “thicker” models of selfhood that track how the organism expects itself to behave and feel over time as well as across different spatial dimensions of the self (Friston 2018; Friston et al. 2018; Deane 2021). By thicker self-models, we refer to those models that pertain to larger spatiotemporal scales that are experienced as more conceptual or narrative aspects of selfhood, which can then loop back to affect more embodied aspects of the self. This hierarchical architecture offers a computational account for how different aspects of selfhood—ranging from interoceptive and embodied to narrative and reflective—can mutually constrain and scaffold one another over time. In line with emerging theories including the three-level model of self-processing (Qin et al. 2020; Wu et al. 2024) and the pattern theory of self (Gallagher 2013), this framework provides a unified mechanism by which bodily, sensory, and mental layers of self-representation interact dynamically to generate the coherent experience of being a unified self. For example, consider someone who suffers from chronic pain. After repeated occurrences of feeling pain without a clear external source (e.g*.* due to internal inflammation), an individual may come to think of themselves as a person who regularly suffers from pain without control over it. This generalized narrative about themselves may then lead them to expect that they will feel recurring pain, which tunes their sensory system to predict these sensations, hence sensitizing them to painful stimuli or even leading them to perceive phantom pain in the absence of any relevant stimulus (Vlaeyen 2015; Van den Bergh et al. 2017; Ongaro and Kaptchuk 2018; Castejón et al. 2024).

This computational description of the self as a hierarchically nested allostatic control model appears to align phenomenologically with Gallagher's “pattern theory of self”. According to Gallagher, the human experience that we call “self” reflects a complex dynamical pattern of self-referential experiences occurring on different spatiotemporal scales, including minimal embodied, minimal experiential, affective, intersubjective, psychological/cognitive, narrative, extended and situated (Gallagher 2013; Gallagher and Daly 2018). Understanding the self as a multiscale predictive model allows us to formalize this dynamical pattern in computational terms and explain the ways in which it may break down under psychedelics (*cf.*  Berkovich-Ohana et al. 2024).

### 1.2. Alterations of the self

According to this active inference view of selfhood, modulating the brain’s allostatic control model can cause changes in the sense of self (Limanowski and Friston 2020). For example, auditory verbal hallucinations may reflect inner speech that is mistakenly attributed to an external source. From an active inference perspective, these misperceptions may result from a failure to accurately predict the internal sensory consequences of one’s own inner speech (Ford and Mathalon 2005; Ford et al. 2007; Safron 2021). The failure to predict the sensory consequences of inner speech at lower-levels may encourage an overweighting of top-down influences from higher-level priors to compensate for the ambiguity in the sensory system (P. Corlett et al. 2019). These top-down priors may then override the incoming sensory data, which may help to explain how auditory verbal hallucinations are shaped by personal history and sociocultural expectations (Luhrmann et al. 2015a, 2015b, 2021; Lifshitz and Luhrmann 2020). Ambiguity at other layers of the hierarchically nested self-model may bring about different changes in the sense of self, including states of ego dissolution, in which more temporally extended, narrative aspects of the self-become disrupted. Psychedelics have been theorized to generate ego dissolution experiences by lowering precision on the high level priors that scaffold precision allocation, causing a collapse in the "temporal thickness" of an agent’s self-model (Deane 2020). According to this view, high level priors in this hierarchical system encode continuous and enduring models of the self and world that persist in light of the fluctuating information on lower sensory levels. Precision of these high level priors supports a sense of continuity of the self. When high-level priors lose their precision, the sense of being a self that is consistent over time disintegrates.

### 1.3. Active inference and the other

Understanding the sense of self as a hierarchically nested process also provides a helpful account of how we model other social beings with whom we are interacting, as well as how we distinguish between our models of self and others. (Friston and Frith 2015) argue that similar computational mechanisms may be responsible for inferring the agency and narrative intentionality of both the self and other. Starting from the premise that agents tend to be similar to the others with whom they are communicating, Friston and Frith suggest that agents use the same generative model to both produce and perceive communication. In this process, the two interacting agents converge on a similar narrative (i.e*.* temporally extended) model of the meaning that is emerging in their interaction, which allows them to produce and interpret communicative behavior in line with this emerging narrative.

Communication, in this view, is the process of generating a shared narrative model that supersedes the agency of either agent alone. There is a separate contextualization process that then distinguishes between those parts of the communication that are self-generated *versus* those that are other-generated. This agentic contextualization process relies on mechanisms of sensory attenuation, which fluctuate based on which of the two agents is currently expressing themselves *versus* listening. When an agent is speaking, they attenuate the sensory consequences of their own actions, which gives them a feeling of agency and facilitates their expression. When an agent is listening, they attend to the sensory signals generated by the other without attenuating these signals, and thus experience the other as agentically expressing themselves. Throughout the whole process, the same computational narrative model is used to express intentions of the self and to interpret the behaviors of the other (Friston et al. 2018; Friston et al. 2021).

Here we propose that the agentic feeling of such interactions is linked to the epistemic richness that is encoded in the shared narrative model (i.e*.* the amount of information/meaning that is novel to the perceiver). In the example of a conversation, there is a constant stream of new information exchanged between two agents, yielding an epistemically rich experience. In contrast, imagine someone listening to a random sequence of sounds. There may be an equal amount of diversity in the low-level perceptual system, but these sounds have less interpretable new information (i.e*.* they are epistemically thinner). As a consequence, it is likely that the listener will attribute less agency to this random sequence of sounds than they would to sounds that carry more novel meaning, such as speech produced by another agent.

Our idea of epistemic richness aligns with an account by Palmer et al. (2015) which proposed that we infer the presence of other intentional minds based on the degree of counterfactual depth that is required to model the intentional states that generated their actions. Counterfactual depth, or richness, refers to the extent to which a generative model can simulate the sensory consequences of multiple possible actions and their outcomes—effectively capturing a wide array of “what if” scenarios. This means that the more complex or ambiguous a given source of sensory input is to infer, the deeper the model must reach into a space of possible counterfactuals to explain it. This model we would then describe as possessing a greater counterfactual depth.

According to Palmer et al. perceiving the mental states of others relies on the same predictive processes that allow us to make inferences about the causes of non-social perceptions. In non-social perception, we generate models to infer the causes of specific sensory experiences. In social perception, however, we must first infer the action of another agent that caused a set of sensations, and then, in a second step, infer the intentional mental state that prompted that action. Thus, perceiving mental states is distinct from perceiving non-mental states because it involves inferring deeper hidden causes. Palmer et al. (2015) argue that agentic presences (i.e*.* other minds) feel more real to us when our models of the causes of their actions are more ambiguous, demanding a greater variety of possible explanations of what state caused that action. They refer to this complexity and ambiguity of the model necessary to infer behavior as the “counterfactual depth” of the model. When a causal state feels harder or more ambiguous to model (i.e*.* has greater counterfactual depth), we tend to infer that it reflects the presence of an intentional, agentic mental state.

In summary, we argue that at a basic embodied level, inferring the presence of agency and discriminating between self and other rely on two distinct yet interacting processes. On the one hand, the presence of intentional agency is inferred based on the epistemic richness or counterfactual depth of a sensory experience: when sensory experiences contain more novel information that is harder to model, this signals to our cognitive system that they were likely intentionally generated by an agentic source. On the other hand, the discrimination of whether that agentic source is our self or another agent relies on whether the sensory signal is attenuated through corollary discharge (i.e. how surprising it is). If the sensation is not attenuated (predicted based on self-initiated motor commands, i.e*.* corollary discharge), then it is more surprising (less expected) to the cognitive system and is therefore attributed to another agent as opposed to the self. In the following section, we apply this nested hierarchical account of agency, self, and self-other discrimination to explain how psychedelics may facilitate experiences of encountering non-self-entities.

## Computational spirits: an active inference account of psychedelic entities

Having laid the groundwork for understanding how the brain makes judgments about agency and distinguishes between self and other, we now build on these insights to propose a neurocomputational model of entity encounters. We have argued above that perceptions are attributed to agentic sources when the perceived epistemic richness and counterfactual depth is high, and that agentic sources are inferred as exogenous when the sensory data is less attenuated through corollary discharge. We use the term *epistemic richness* to describe stimuli or percepts that introduce a high degree of novel or previously inaccessible information into conscious awareness. When such information is also difficult to explain or predict—i.e*.* when it requires deeper generative models to account for its causes—these models have high *counterfactual depth*. Drawing on Palmer et al. (2015), we argue that the brain uses the properties of epistemic richness and counterfactual depth as cues for inferring intentional agency. In other words, when an experience is unusually information-rich and difficult to model causally, the brain is inclined to resolve this ambiguity by postulating an agentic source. Under psychedelics, the relaxation of high-level priors may increase the probability of such inferences, especially when combined with reduced sensory attenuation that leads to a feeling of external origin.

**Figure 1:**
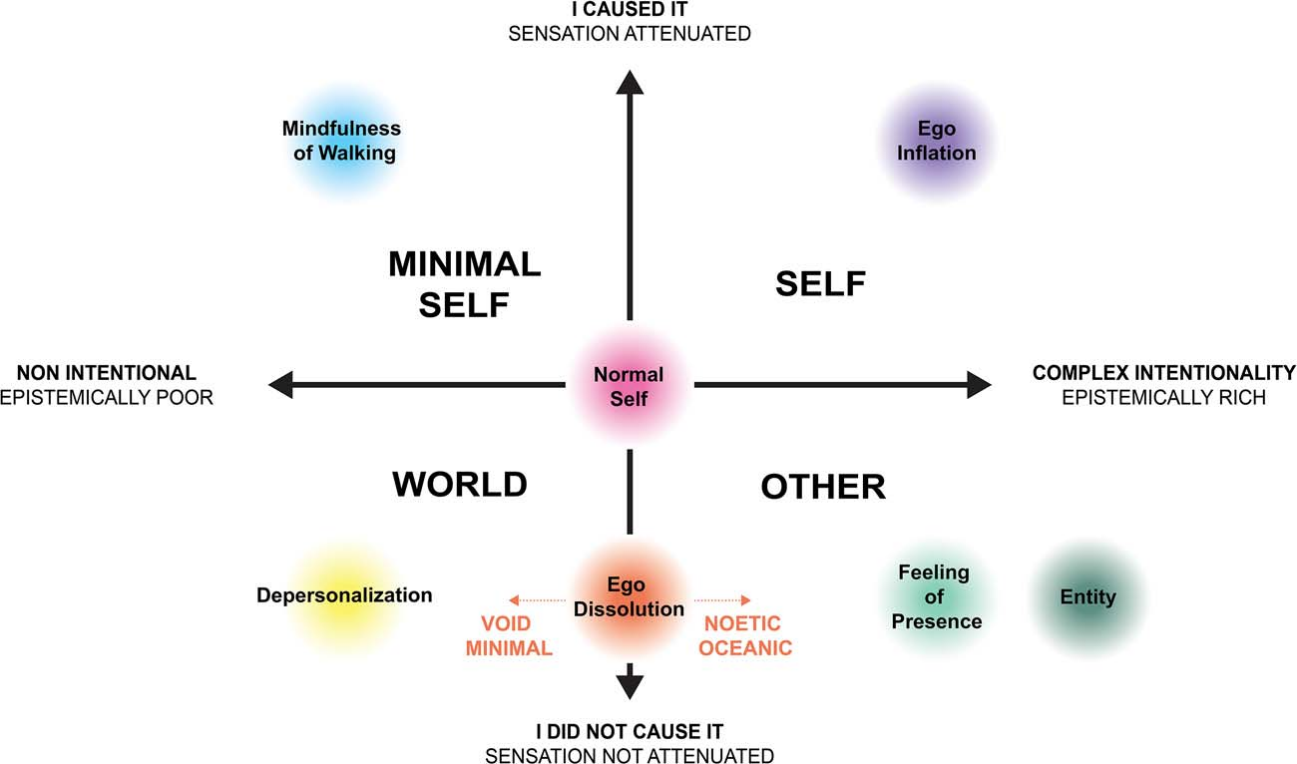
Two-dimensional model of self–other discrimination and agency attribution in altered states of consciousness. The vertical axis represents the degree of sensory attenuation through corollary discharge: Attenuated sensations are attributed to self-generated causes (*I caused it*), while un-attenuated sensations are attributed to exogenous sources (*I did not cause it*). The horizontal axis represents the epistemic richness (or counterfactual depth) of experience—That is, the degree of novel, meaningful information inferred from sensory input—Ranging from epistemically poor and non-intentional experiences to epistemically rich perceptions implying complex intentionality. Here, epistemic richness refers not to momentary sensory surprise but to the complexity of the intentional model implied by the experience—Richer for agentic or personified models, poorer for minimal or purely sensory ones. According to this model, self-experiences involve attenuated sensations attributed to complex intentional self-models; minimal self-experiences involve attenuated sensations with low epistemic richness, such as mindfulness of walking; other experiences arise when sensations are un-attenuated yet epistemically rich, producing attributions to external agents or entities; and world experiences involve un-attenuated, epistemically poor sensations, as in severe depersonalization. Ego dissolution spans the lower middle axis, with the *noetic-oceanic* variant retaining epistemic richness and the *void-minimal* variant lacking both self-attribution and content. The normal self lies at the center, reflecting typical baseline functioning across both dimensions.

Following this model, here we argue that entity encounters arise when sensory contents are both epistemically rich—prompting the inference of intentional agency—and un-attenuated (exogenous), leading to their attribution to an external, non-self-source. This relationship can be formalized as a two-dimensional space defined by epistemic richness and sensory attenuation (Figure 1).

Below we will explain how psychedelics may facilitate such experiences of entities by synthesizing the active inference perspective on agency and self-other discrimination with the REBUS model of psychedelic brain function. We argue that the loosening of top-down constraints from high-level priors in the hierarchical self-world model may increase the proliferation of epistemically rich content (either suppressed unconscious content or previously unnoticed patterns in the world) and alter sensory attenuation mechanisms in a way that pushes the system to attribute agency exogenously. This then leads to experiences of epistemically rich, non-self-entities.

First, we have argued that the feeling of agency is associated with the epistemic richness and counterfactual depth of experience. The epistemic richness of an experience is the degree to which an experience reveals previously unknown information to consciousness. In ordinary waking states, sensations, beliefs, and experiences that contradict the brain’s current model of the world are experienced as particularly salient. However, recent work on avoidant mental actions suggests that when the difference becomes overly jarring or threatening to the current model of the world, the intensity of prediction error can lead to an influx of negative affect that may prevent the organism from acting efficiently, similar to the idea of cognitive dissonance (Deane et al. 2024). To prevent this overwhelming flood of negative affect, experiences that are too shocking to the current model of the world may be suppressed to maintain and protect this old model rather than update it. In these situations, the weighting of the prior model suppresses the prediction error arising from the contradictory sensory data. Recent work in active inference has described this as a process of “avoidant mental action” or “motivated inattention,” providing a computational account of classical psychoanalytic defense mechanisms such as repression. This “computational unconscious” model describes how subpersonal computational processes may adaptively control the contents of experience to avoid challenging mental content, even at the cost of reduced fidelity to the external world (Deane et al. 2024). In this light, the resistance to contradictory information in ordinary waking states reflects a regime of high prior precision that overdetermines perception, minimizing disruptive updates. In contrast, in psychedelic and other altered states of consciousness, the precision of these priors may be relaxed, allowing for greater epistemic flexibility and salience of previously suppressed or contradictory information. These complementary dynamics—suppression under strong priors and salience under weak priors—are both expressions of the same underlying inferential machinery.

During the psychedelic state, according to the REBUS account, high-level models of the world are disrupted and relaxed. This relaxation of high-level models may reduce the need and/or capacity of the cognitive system to adapt its experiences to conform to a rigid expectation of how the world is. By reducing motivated inattention and avoidance mechanisms, psychedelics may then allow previously repressed unconscious mental contents or suppressed sensory stimuli to rise into consciousness. Previously, these perceptual contents were suppressed because the brain was not able to reconcile them easily with its current model of the world. Now that the brain has lifted its suppression under psychedelics, perceptions that were suppressed (specifically because they were hard to integrate with the current world model) become available and the brain is forced to infer the causes of these perceptions. However, the brain struggles to model the hidden causes of these perceptions, because all of the potential explanatory models seem to contradict in some way with the current world model. Thus, the brain is forced to adopt a wider diversity of potential models to infer the underlying hidden causes of these perceptions, and to hold each of these potential models with less confidence and more ambiguity. This corresponds to a greater epistemic richness of these previously suppressed contents (i.e*.* more novelty) and greater counterfactual depth of the models necessary to explain their hidden causes. Because these perceptions come along with greater epistemic richness and counterfactual depth, they are more likely to be inferred as originating from intentional agentic presences. Furthermore, since counterfactual depth entails modeling a wider range of possible meanings, outcomes, and intentional structures, the inferred agents may appear to carry complex knowledge, purpose, or insight. As a result, the content of these experiences is more likely to be interpreted as morally or metaphysically significant, giving rise to impressions of guidance, revelation, or profound truth, or a “mystical glow.”

The above account of increased epistemic richness and counterfactual depth helps to explain why psychedelics may lead to experiences of agentic presence. However, this explanation does not yet explain why psychedelic entities feel as if they are external/different from the person experiencing them, particularly in the case of entities that are experienced in the imaginal realm as opposed to the material world. Why would such agentic presences be experienced as distinct from the self if they occur in the private space of one’s mind? Normally when we experience a complex thought or mental image, it might feel agentic, yet we experience it as originating from our own self. In the case of imaginal entities, the perception occurs in the mind space and yet feels as if it has its own independent agency. Above, we discussed sensory attenuation as a central mechanism that the brain uses to discern if an experience is caused from within (endogenous) or by an external source (exogenous). The more precisely an experience is predicted to arise as a consequence of one's own action (including mental action), the more likely that experience is to be perceived as endogenously caused. According to REBUS, psychedelics reduce the precision of top-down signaling in the brain. This likely disrupts sensory attenuation mechanisms, which rely on comparing incoming sensations to the predicted sensory consequences of one’s own actions. When these mechanisms are compromised, the brain is more likely to interpret the source of experience as exogenous rather than self-generated. These alterations may also disinhibit neural systems involved in social cognition, such as the medial temporal lobe, superior temporal sulcus, and TPJ—regions implicated in theory of mind and the inference of agency (Frith, 2000; Aichhorn et al. 2009; Koster-Hale & Saxe, 2013). Supporting this view, Michael et al. (2021) propose that under DMT, the breakdown of intrinsic network organization—including disintegration of the DMN and dysregulation of temporal lobe activity—may release an evolutionarily conserved tendency toward hyperactive agency detection (see also, Winkelman 2019). These proposals converge with our model in suggesting that disrupted top-down constraints and altered theory-of-mind processes may render the brain more likely to attribute agency to ambiguous or richly patterned sensory information.

In summary, we propose that entity encounters are facilitated by the co-occurrence of two processes: an increase in the epistemic richness/counterfactual depth of experience combined with an increased tendency to attribute perceptions to exogenous sources, as captured by a two-dimensional model of self–other discrimination and agency attribution (Figure 1). During the (relatively) uncertain (i.e. entropic) state under psychedelics, the brain attempts to generate the most accurate model of sensory experience. We propose that entity encounters emerge as the cognitive system finds the “best-guess” approximation, or generative model, to explain the volatile sensory data, rather than overdetermining perception through strong *a priori* models of the world as in the usual waking state. Under psychedelics, the perceptual data may be both more epistemically rich/counterfactually deep and less reliably attenuated by sensory predictions. Thus, the brain is tuned to infer the presence of intentional agency (due to increased epistemic richness/counterfactual depth) and to attribute this agency to a source other than the self (due to less reliable sensory attenuation).

We wish to acknowledge several limitations of our model. First, as our model is specifically targeted towards entity encounters in the psychedelic context, it remains an open question if, and to what extent, the mechanisms outlined here generalize to explain entity experiences in contexts outside the psychedelic state. Even within the psychedelic context, our model focuses specifically on the phenomenology and neurophysiology of classical tryptamines such as DMT and may not capture the full diversity of entity encounters across substances and contexts. Second, while our model is grounded in the active inference framework, we do not provide a formal mathematical implementation of the model. This limits the testability and precision of our claims. Future work should develop generative models that simulate the proposed mechanisms under varying degrees of sensory attenuation, prior precision, and counterfactual depth. Third, entity encounters are not uniformly observed across all serotonergic psychedelics. For instance, 5-MeO-DMT, despite its structural similarity to N,N-DMT, often induces experiences marked by non-duality or ego dissolution rather than the presence of autonomous entities. This contrast highlights the need for further research into the substance-specific neurocomputational profiles that modulate whether—and how—agentic presences are inferred (Michael, 2021).

Despite these limitations, future empirical work could further validate our theoretical model, contingent on the development of good empirical measures of counterfactual depth and sensory attenuation. While we do not have a direct neurophysiological correlate of counterfactual depth, we might envision a situation where a participant interacts with a simulated object/agent that is designed to possess a more or less complex repertoire of action possibilities—for example, in a simple virtual game. By modulating the complexity of the object/agent’s action repertoire and measuring the participant’s neural or behavioral reactions to those actions, the experiment could estimate the counterfactual depth of the participant’s model of the object/action. We would predict that on psychedelics, participants would be less surprised if a non-intentional object started responding in a way that implied intentionality, given that their model of objects would already tend to be more counterfactually deep. In addition to counterfactual depth, we have also argued that entity experiences are associated with a disruption of sensory attenuation mechanisms. Thus, under the influence of psychedelics, and perhaps particularly during entity episodes *per se*, we would predict a less attenuated response in somatosensory cortex in response to self-initiated movements.

### The cultural patterning of entity experiences

In line with the REBUS model, we have attempted to explain entity experiences *via* decreased precision in high-level priors, leading to an increase in previously suppressed information from either sensory inputs or the imagination (including what some might call the unconscious). REBUS provides an elegant account of how the relaxation of the brain’s high-level beliefs/models of the self and world may allow for a greater diversity and richness of experience to be released under psychedelics. However, the relaxation of prior beliefs does not seem to account, in itself, for the fact that the specific contents of psychedelic experiences are profoundly shaped by the cultural contexts in which these experiences are embedded. If psychedelics mostly relax prior beliefs and models of the world, why does this lead to the emergence of visionary experiences and agentic encounters that are deeply shaped by local beliefs and expectations? (Lifshitz et al. 2018).

Using ethnographic data collected in a shamanic center of the Peruvian Amazon, Dupuis (2022b) has argued that cultural background and social interactions organize not only the meaning-making surrounding the psychedelic experience, but also its very phenomenological content. His ethnographic observations suggest that the processes that lead individuals to interpret ambiguous visual and auditory stimuli as an identifiable entity are highly plastic and seem to be gradually modified and patterned by the social environment. He and other scholars have proposed that encountering entities is an active process, in which people learn to attend to their experience in a way that brings forth culturally salient perceptions (*cf.*  Luhrmann et al. 2023). Dupuis describes several mechanisms that may underpin this learning process—which he calls the “socialization of hallucinations”—including the education of attention, the categorization of perceptions, and the shaping of emotions and expectations. The discursive and ritual interactions, as well as the iconographic elements that frame the psychedelic experience, seem to shape expectations in a way that “socializes” the phenomenological content of the experience.

Conceptually, this gives credence to an interpretation according to which people "search" for experiences and descriptors of their experiences that match cultural expectations. Under psychedelics, the relaxation of higher-order constraints on perception may force the system to make a best guess to interpret the sources that cause the perceptual data. The individual and contextual patterning of entity experiences may reflect the diversity of information that people have stored over the course of their lives to interpret noisy data. For example, recent work suggests that hallucination-like alterations in experience can partially be accounted for by an over-weighting of top-down priors in response to ambiguity in the lower levels of sensory prediction (Corlett and Powers 2018; Safron 2025). Recent models have extrapolated this approach to argue that much of our everyday cognition and perception is constrained by top-down priors that are transmitted to us through cultural expectations, “regimes of attention”, or “cultural affordances” in our social environments (Ramstead et al. 2016; Veissière et al. 2020). Extending this idea, findings show that the patterns we encounter in our lived environments seem to “imprint” our sensory systems to evoke similarly patterned visions during psychedelic experiences (Garel et al. 2023). For example, in one study almost half of white westerners who took ibogaine reported prominent visual hallucinations of television screens (Heink et al. 2017). In contrast, ethnographic accounts of Ibogaine use in African Bitwi communities rarely include screens and tend to center around experiences of ancestors and immersion in landscapes (Fernandez 2019).

To explain the strong influence of cultural patterning on psychedelic experience, we turn to recent computational accounts suggesting that psychedelics may involve not only the relaxing of higher-order beliefs as proposed by REBUS, but also the strengthening of prior expectations. According to this more encompassing ALBUS, or ALtered Beliefs Under pSychedelics, framework (Safron 2025), psychedelics may lead to both direct and indirect strengthening and/or relaxation of beliefs at different levels in the cognitive hierarchy as a function of substance and dose but also set and setting, including sociocultural context. Through the release of precision in higher-order neural populations, which typically constrain activity in lower levels of the generative model, new ordered firing patterns in middle and low level neural populations may emerge. The patterns that emerge from these mid- and low-levels are likely to be partially constituted by evolved, “universal” tendencies in human cognition (e.g. the tendency to see faces in noise, or to attribute agency to motion resembling biological movement), but also shaped by priors learned through each individual’s particular social context and lived environment (e.g. the cultural expectation that one will encounter a particular kind of spirit, or the imprinting of television screens on one’s visual priors). Importantly, counterfactual depth does not oppose the influence of strong priors but reflects the complexity of the internal model used to explain a percept. A psychedelic experience can be shaped by culturally or evolutionarily reinforced expectations (i.e. strong priors) in a way that is either counterfactually deep or shallow. For example, if a Christian takes a psychedelic and experiences a vision of a cross, this experience would likely be constrained by strong priors, yet counterfactually shallow. If, instead, they see an image of Christ speaking to them with a feeling of independent intentionality, this would reflect an experience that is not only constrained by strong priors but also high in counterfactual depth.

According to the ALBUS model, whether specific priors are strengthened or relaxed is determined by the level of stimulation of 5-HT2a receptors on deep pyramidal neurons, which are thought to be largely responsible for encoding Bayesian priors in predictive processing models. Therefore, in addition to belief-relaxation, it is important to consider alternative mechanistic hypotheses in which prior expectations received through cultural and personal context may be directly strengthened under the influence of psychedelics, potentially leading to the manifestation of culturally salient entities. While the precise mechanisms by which certain priors become strengthened under psychedelics remain to be elucidated, acknowledging this possibility opens an important avenue for future research—suggesting that, alongside general belief relaxation, some culturally and personally salient expectations may gain relative prominence and shape the specific content of non-self-entity encounters.

From a different ontological perspective, this model could allow for the possibility that the perceived entities may in fact be present, regardless of one's expectations about them. Even in cultures that strongly sanction the existence of entities, anthropological evidence suggests that people still struggle to overcome skepticism and doubt, especially given that most have rarely experienced an entity with their own senses (Boyer 2013; Luhrmann 2022; Dupuis 2022b). In this case, attending with less rigid priors may allow the perceptual system to relax its expectations against the existence of the entities and thus allow the sensory signals caused by their presence to become more salient and reach the threshold of conscious perception.

## Conclusion

In this paper, we have presented a neurocomputational model of psychedelic entity encounters. Our model begins from empirical and theoretical work suggesting that social cognition relies on the brain's capacity to build and maintain models of the self-organism and of the mental states of other agents in the environment. We propose that in the psychedelic state, sensory consequences of mental actions become radically unpredictable, altering two key processes involved in making inferences about other agents. First, the psychedelic state relaxes higher order constraints on perception, leading to an influx of previously suppressed perceptual content from both the imagination and the sensory environment. This causes an increase in the epistemic richness or the counterfactual depth of the perceived signals, and thus indicates to the brain that these signals were likely caused by an intentional agent. Second, by relaxing higher-order perceptual models, the psychedelic state reduces the precision of top-down sensory attenuation mechanisms, which are typically used to discern the self as the cause of sensory signals. As a consequence, the brain may tend to infer that its perceptions are caused exogenously, by something other than the self. Taking both of these mechanisms together, we argue that psychedelics tune the brain to infer the presence of agentic, non-self-entities. Finally, our model integrates recent work on the cultural patterning of psychedelic experiences by arguing that cultural learning shapes not only high-level, but also mid-level priors. When psychedelics reduce the precision of higher-order beliefs, the culturally patterned priors in the middle layers may exert a more powerful influence on how ambiguous sensory inputs are resolved—helping to determine the specific forms that agentic entities take.

## Data Availability

No new data were generated or analyzed in support of this research.
